# Bis[(2-amino­phen­yl)methanol-κ^2^ *O*,*N*]bis­(nitrato-κ*O*)manganese(II)

**DOI:** 10.1107/S1600536810030114

**Published:** 2010-08-04

**Authors:** Majid Esmhosseini, Shahrzad Maleki

**Affiliations:** a Department of Chemistry, University of Urmiyeh, Urmyieh, Iran

## Abstract

In the title compound, [Mn(NO_3_)_2_(C_7_H_9_NO)_2_], the Mn^II^ atom (site symmetry 2) is coordinated by two *N*,*O*-bidentate (2-amino­phen­yl)methanol ligands and two monodentate nitrate anions in a distorted *cis*-MnN_2_O_4_ octa­hedral coordination geometry. In the crystal, N—H⋯O, O—H⋯O and C—H⋯O hydrogen bonds help to establish the packing.

## Related literature

For structures involving the same ligand with other metal ions, see: Bandoli *et al.* (2002 ▶); Lewiriski *et al.* (1998 ▶).
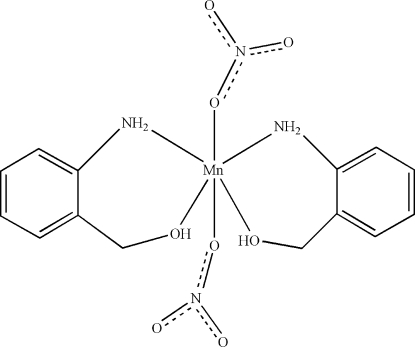


## Experimental

### 

#### Crystal data

[Mn(NO_3_)_2_(C_7_H_9_NO)_2_]
*M* *_r_* = 425.26
Orthorhombic, 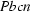

*a* = 23.374 (2) Å
*b* = 10.1929 (12) Å
*c* = 7.3336 (6) Å
*V* = 1747.2 (3) Å^3^
*Z* = 4
Mo *K*α radiation
μ = 0.81 mm^−1^
*T* = 120 K
0.40 × 0.10 × 0.06 mm

#### Data collection

Bruker SMART CCD diffractometer
Absorption correction: multi-scan (*SADABS*; Bruker, 1998 ▶) *T*_min_ = 0.907, *T*_max_ = 0.955
6668 measured reflections
2335 independent reflections
1928 reflections with *I* > 2σ(*I*)
*R*_int_ = 0.046

#### Refinement

*R*[*F* ^2^ > 2σ(*F* ^2^)] = 0.038
*wR*(*F* ^2^) = 0.071
*S* = 1.10
2335 reflections
135 parameters
H atoms treated by a mixture of independent and constrained refinement
Δρ_max_ = 0.31 e Å^−3^
Δρ_min_ = −0.32 e Å^−3^

### 

Data collection: *SMART* (Bruker, 1998 ▶); cell refinement: *SAINT* (Bruker, 1998 ▶); data reduction: *SAINT*; program(s) used to solve structure: *SHELXTL* (Sheldrick, 2008 ▶); program(s) used to refine structure: *SHELXTL*; molecular graphics: *ORTEP-3* (Farrugia, 1997 ▶); software used to prepare material for publication: *WinGX* (Farrugia, 1999 ▶).

## Supplementary Material

Crystal structure: contains datablocks I, global. DOI: 10.1107/S1600536810030114/hb5561sup1.cif

Structure factors: contains datablocks I. DOI: 10.1107/S1600536810030114/hb5561Isup2.hkl

Additional supplementary materials:  crystallographic information; 3D view; checkCIF report

## Figures and Tables

**Table 1:** Selected bond lengths (Å)

Mn1—N1	2.2469 (15)
Mn1—O1	2.2041 (13)
Mn1—O2	2.2203 (12)

**Table 2:** Hydrogen-bond geometry (Å, °)

*D*—H⋯*A*	*D*—H	H⋯*A*	*D*⋯*A*	*D*—H⋯*A*
N1—H1*C*⋯O3^i^	0.87 (2)	2.16 (2)	2.9775 (19)	156 (2)
N1—H1*D*⋯O4^ii^	0.90 (3)	2.15 (3)	3.037 (2)	169 (2)
O1—H1*E*⋯O2^iii^	0.82 (3)	1.88 (3)	2.6937 (17)	176 (3)
C1—H1*B*⋯O4^ii^	0.97	2.60	3.479 (2)	151

Symmetry codes: (i) 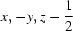; (ii) 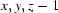; (iii) 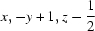.

**Fig. 1.**
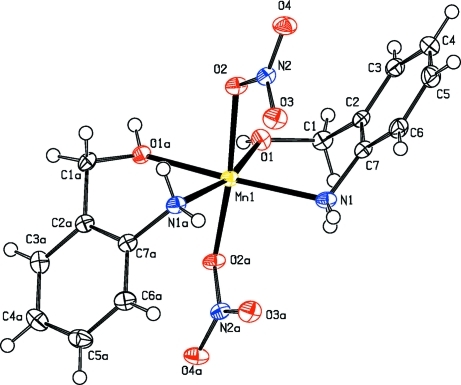
The molecular structure of (I) with displacement ellipsoids drawn at the 50% probability level. Atoms with suffix a are generated by (1–x, y, 1/2–z).

**Fig. 2.**
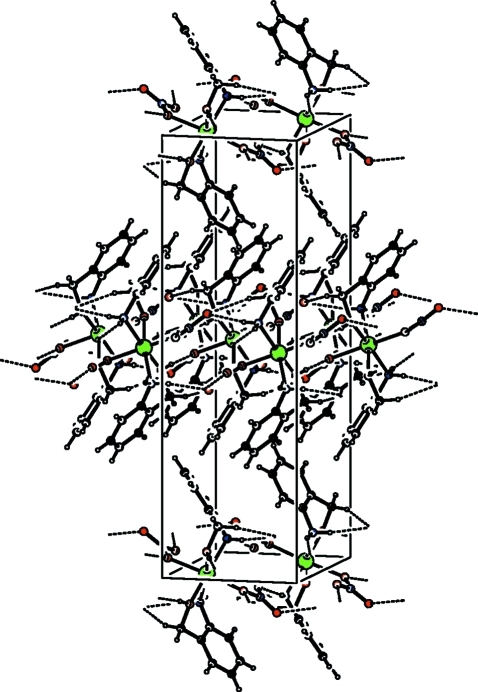
Unit-cell packing diagram for (I). Hydrogen bonds are shown as dashed lines.

## supplementary crystallographic information

### Comment

(2-Aminophenyl)methanol is a bidentate ligand ligand. There are only two complexes with this ligand that have been prepared: those of Re (Bandoli *et al.*, 2002) and Al (Lewiriski *et al.* 1998). We report herein the synthesis and crystal structure of the title compound, (I).

The asymmetric unit of the title compound, Fig. 1, contains half molecule. The Mn^II^ atom is six-coordinated in distorted hexagonal configurations by two N and two O atoms from two (2-aminophenyl)methanol ligand and two O atoms from two nitrate anions. The Mn—O and Mn—N bond lengths and angles are collected in Table 1.

Intermolecular N—H···O, O—H···O and C—H···O hydrogen bonding may stabilize the structure, (Table 2, Fig. 2).

### Experimental

A solution of (2-aminophenyl)methanol (0.25 g, 2.00 mmol) in methanol (10 ml) was added to a solution of Mn(NO_3_)_2_.4H_2_O (0.25 g, 1.00 mmol) in methanol (10 ml) and the resulting colorless solution was stirred for 20 min at 313 K. This solution was left to evaporate slowly at room temperature. After one week, colorless needles of (I) were isolated (yield 0.33 g, 77.6%).

### Refinement

The N- and O-bound H atoms were located in a difference map and freely refined.  All C-bound H atoms were positioned geometrically, with C—H = 0.93Å and constrained to ride on their parent atoms, with *U*_iso_(H) = 1.2*U*_eq_(C).

### Crystal data

**Table:**

[Mn(NO_3_)_2_(C_7_H_9_NO)_2_]	*F*(000) = 876
*M**_r_* = 425.26	*D*_x_ = 1.617 Mg m^−^^3^
Orthorhombic, *P**b**c**n*	Mo *K*α radiation, λ = 0.71073 Å
Hall symbol: -P 2n 2ab	Cell parameters from 1154 reflections
*a* = 23.374 (2) Å	θ = 2.2–29.2°
*b* = 10.1929 (12) Å	µ = 0.81 mm^−^^1^
*c* = 7.3336 (6) Å	*T* = 120 K
*V* = 1747.2 (3) Å^3^	Block, colorless
*Z* = 4	0.40 × 0.10 × 0.06 mm

### Data collection

**Table:**

Bruker SMART CCD diffractometer	2335 independent reflections
Radiation source: fine-focus sealed tube	1928 reflections with *I* > 2σ(*I*)
graphite	*R*_int_ = 0.046
phi and ω scans	θ_max_ = 29.2°, θ_min_ = 2.2°
Absorption correction: multi-scan (*SADABS*; Bruker, 1998)	*h* = −31→23
*T*_min_ = 0.907, *T*_max_ = 0.955	*k* = −13→10
6668 measured reflections	*l* = −9→9

### Refinement

**Table:**

Refinement on *F*^2^	Primary atom site location: structure-invariant direct methods
Least-squares matrix: full	Secondary atom site location: difference Fourier map
*R*[*F*^2^ > 2σ(*F*^2^)] = 0.038	Hydrogen site location: inferred from neighbouring sites
*wR*(*F*^2^) = 0.071	H atoms treated by a mixture of independent and constrained refinement
*S* = 1.10	*w* = 1/[σ^2^(*F*_o_^2^) + (0.017*P*)^2^ + 1.3224*P*] where *P* = (*F*_o_^2^ + 2*F*_c_^2^)/3
2335 reflections	(Δ/σ)_max_ = 0.007
135 parameters	Δρ_max_ = 0.31 e Å^−^^3^
0 restraints	Δρ_min_ = −0.32 e Å^−^^3^

### Special details

**Table:**

Geometry. All e.s.d.'s (except the e.s.d. in the dihedral angle between two l.s. planes) are estimated using the full covariance matrix. The cell e.s.d.'s are taken into account individually in the estimation of e.s.d.'s in distances, angles and torsion angles; correlations between e.s.d.'s in cell parameters are only used when they are defined by crystal symmetry. An approximate (isotropic) treatment of cell e.s.d.'s is used for estimating e.s.d.'s involving l.s. planes.
Refinement. Refinement of *F*^2^ against ALL reflections. The weighted *R*-factor *wR* and goodness of fit *S* are based on *F*^2^, conventional *R*-factors *R* are based on *F*, with *F* set to zero for negative *F*^2^. The threshold expression of *F*^2^ > σ(*F*^2^) is used only for calculating *R*-factors(gt) *etc*. and is not relevant to the choice of reflections for refinement. *R*-factors based on *F*^2^ are statistically about twice as large as those based on *F*, and *R*- factors based on ALL data will be even larger.

### Fractional atomic coordinates and isotropic or equivalent isotropic displacement parameters (Å^2^)

**Table:**

	*x*	*y*	*z*	*U*_iso_*/*U*_eq_	
C1	0.61659 (7)	0.43167 (16)	0.1127 (2)	0.0177 (3)	
H1A	0.6380	0.5132	0.1158	0.021*	
H1B	0.6137	0.4033	−0.0132	0.021*	
C2	0.64736 (7)	0.32879 (16)	0.2234 (2)	0.0160 (3)	
C3	0.69730 (8)	0.36086 (18)	0.3180 (2)	0.0208 (3)	
H3	0.7119	0.4455	0.3095	0.025*	
C4	0.72554 (8)	0.26846 (19)	0.4247 (3)	0.0248 (4)	
H4	0.7586	0.2911	0.4876	0.030*	
C5	0.70376 (8)	0.14206 (19)	0.4363 (3)	0.0248 (4)	
H5	0.7221	0.0802	0.5087	0.030*	
C6	0.65484 (8)	0.10717 (17)	0.3408 (2)	0.0197 (3)	
H6	0.6411	0.0217	0.3473	0.024*	
C7	0.62624 (7)	0.20036 (16)	0.2348 (2)	0.0162 (3)	
N1	0.57317 (7)	0.16868 (14)	0.1479 (2)	0.0168 (3)	
H1D	0.5731 (10)	0.194 (2)	0.030 (4)	0.033 (6)*	
H1C	0.5657 (10)	0.085 (2)	0.156 (3)	0.027 (6)*	
N2	0.55303 (6)	0.21326 (14)	0.61414 (18)	0.0165 (3)	
O1	0.55977 (5)	0.45233 (12)	0.18714 (17)	0.0181 (2)	
H1E	0.5502 (11)	0.522 (3)	0.141 (4)	0.046 (8)*	
O2	0.53386 (6)	0.31604 (10)	0.53077 (16)	0.0184 (3)	
O3	0.53757 (6)	0.10431 (11)	0.55841 (18)	0.0241 (3)	
O4	0.58609 (6)	0.22845 (13)	0.74423 (18)	0.0249 (3)	
Mn1	0.5000	0.29131 (3)	0.2500	0.01412 (9)	

### Atomic displacement parameters (Å^2^)

**Table:**

	*U*^11^	*U*^22^	*U*^33^	*U*^12^	*U*^13^	*U*^23^
C1	0.0222 (8)	0.0131 (7)	0.0179 (8)	−0.0029 (6)	0.0022 (7)	0.0018 (6)
C2	0.0186 (7)	0.0156 (7)	0.0137 (8)	0.0016 (6)	0.0017 (6)	−0.0019 (6)
C3	0.0197 (8)	0.0212 (8)	0.0216 (8)	−0.0015 (7)	0.0024 (7)	−0.0055 (7)
C4	0.0196 (8)	0.0306 (10)	0.0243 (8)	0.0028 (8)	−0.0040 (7)	−0.0056 (8)
C5	0.0244 (9)	0.0276 (10)	0.0224 (8)	0.0090 (8)	−0.0023 (7)	0.0006 (8)
C6	0.0237 (8)	0.0152 (7)	0.0203 (8)	0.0043 (7)	0.0000 (7)	−0.0005 (7)
C7	0.0191 (7)	0.0151 (7)	0.0143 (7)	0.0014 (6)	0.0016 (6)	−0.0017 (6)
N1	0.0230 (7)	0.0109 (6)	0.0165 (7)	−0.0018 (5)	−0.0026 (6)	−0.0005 (5)
N2	0.0213 (7)	0.0139 (6)	0.0144 (6)	0.0013 (6)	0.0004 (5)	0.0012 (5)
O1	0.0204 (6)	0.0102 (5)	0.0238 (6)	−0.0002 (5)	−0.0008 (5)	0.0045 (5)
O2	0.0288 (6)	0.0087 (5)	0.0177 (6)	0.0011 (5)	−0.0042 (5)	0.0014 (4)
O3	0.0316 (7)	0.0103 (5)	0.0305 (7)	−0.0017 (5)	−0.0047 (6)	0.0007 (5)
O4	0.0308 (6)	0.0276 (7)	0.0163 (6)	0.0021 (5)	−0.0072 (6)	0.0004 (6)
Mn1	0.01774 (16)	0.00979 (14)	0.01484 (16)	0.000	−0.00214 (14)	0.000

### Geometric parameters (Å, °)

**Table:**

C1—O1	1.451 (2)	C7—N1	1.431 (2)
C1—C2	1.509 (2)	Mn1—N1	2.2469 (15)
C1—H1A	0.9700	N1—H1D	0.90 (3)
C1—H1B	0.9700	N1—H1C	0.87 (2)
C2—C3	1.397 (2)	N2—O3	1.2372 (19)
C2—C7	1.402 (2)	N2—O4	1.2375 (18)
C3—C4	1.391 (3)	N2—O2	1.2931 (18)
C3—H3	0.9300	Mn1—O1	2.2041 (13)
C4—C5	1.388 (3)	O1—H1E	0.82 (3)
C4—H4	0.9300	Mn1—O2	2.2203 (12)
C5—C6	1.387 (3)	Mn1—O1^i^	2.2041 (13)
C5—H5	0.9300	Mn1—O2^i^	2.2202 (12)
C6—C7	1.398 (2)	Mn1—N1^i^	2.2469 (15)
C6—H6	0.9300		
			
O1—C1—C2	109.56 (13)	Mn1—N1—H1D	99.2 (16)
O1—C1—H1A	109.8	C7—N1—H1C	111.3 (15)
C2—C1—H1A	109.8	Mn1—N1—H1C	111.6 (15)
O1—C1—H1B	109.8	H1D—N1—H1C	110 (2)
C2—C1—H1B	109.8	O3—N2—O4	123.32 (14)
H1A—C1—H1B	108.2	O3—N2—O2	118.03 (13)
C3—C2—C7	118.92 (15)	O4—N2—O2	118.65 (14)
C3—C2—C1	120.20 (15)	C1—O1—Mn1	123.42 (10)
C7—C2—C1	120.88 (15)	C1—O1—H1E	102.7 (19)
C4—C3—C2	121.16 (17)	Mn1—O1—H1E	124.1 (19)
C4—C3—H3	119.4	N2—O2—Mn1	118.03 (10)
C2—C3—H3	119.4	O1^i^—Mn1—O1	83.74 (7)
C5—C4—C3	119.26 (17)	O1^i^—Mn1—O2^i^	83.31 (5)
C5—C4—H4	120.4	O1—Mn1—O2^i^	86.99 (5)
C3—C4—H4	120.4	O1^i^—Mn1—O2	86.99 (5)
C6—C5—C4	120.63 (17)	O1—Mn1—O2	83.31 (5)
C6—C5—H5	119.7	O2^i^—Mn1—O2	166.96 (6)
C4—C5—H5	119.7	O1^i^—Mn1—N1^i^	82.07 (5)
C5—C6—C7	120.05 (17)	O1—Mn1—N1^i^	165.12 (5)
C5—C6—H6	120.0	O2^i^—Mn1—N1^i^	95.79 (5)
C7—C6—H6	120.0	O2—Mn1—N1^i^	91.45 (5)
C6—C7—C2	119.96 (15)	O1^i^—Mn1—N1	165.11 (5)
C6—C7—N1	120.58 (15)	O1—Mn1—N1	82.07 (5)
C2—C7—N1	119.31 (14)	O2^i^—Mn1—N1	91.45 (5)
C7—N1—Mn1	112.68 (10)	O2—Mn1—N1	95.79 (5)
C7—N1—H1D	111.3 (16)	N1^i^—Mn1—N1	112.40 (8)
			
O1—C1—C2—C3	−117.48 (17)	O4—N2—O2—Mn1	159.69 (11)
O1—C1—C2—C7	61.89 (19)	C1—O1—Mn1—O1^i^	−175.17 (15)
C7—C2—C3—C4	−1.1 (3)	C1—O1—Mn1—O2^i^	−91.58 (12)
C1—C2—C3—C4	178.31 (16)	C1—O1—Mn1—O2	97.15 (12)
C2—C3—C4—C5	0.4 (3)	C1—O1—Mn1—N1^i^	167.15 (17)
C3—C4—C5—C6	0.9 (3)	C1—O1—Mn1—N1	0.31 (12)
C4—C5—C6—C7	−1.4 (3)	N2—O2—Mn1—O1^i^	148.15 (12)
C5—C6—C7—C2	0.7 (2)	N2—O2—Mn1—O1	−127.80 (12)
C5—C6—C7—N1	−174.78 (16)	N2—O2—Mn1—O2^i^	−169.96 (11)
C3—C2—C7—C6	0.5 (2)	N2—O2—Mn1—N1^i^	66.17 (12)
C1—C2—C7—C6	−178.84 (15)	N2—O2—Mn1—N1	−46.52 (12)
C3—C2—C7—N1	176.07 (15)	C7—N1—Mn1—O1^i^	69.1 (2)
C1—C2—C7—N1	−3.3 (2)	C7—N1—Mn1—O1	51.34 (11)
C6—C7—N1—Mn1	117.00 (14)	C7—N1—Mn1—O2^i^	138.10 (11)
C2—C7—N1—Mn1	−58.50 (17)	C7—N1—Mn1—O2	−31.05 (12)
C2—C1—O1—Mn1	−49.35 (17)	C7—N1—Mn1—N1^i^	−125.03 (12)
O3—N2—O2—Mn1	−19.78 (18)		

Symmetry codes: (i) −*x*+1, *y*, −*z*+1/2.

### Hydrogen-bond geometry (Å, °)

**Table:**

*D*—H···*A*	*D*—H	H···*A*	*D*···*A*	*D*—H···*A*
N1—H1C···O3^ii^	0.87 (2)	2.16 (2)	2.9775 (19)	156 (2)
N1—H1D···O4^iii^	0.90 (3)	2.15 (3)	3.037 (2)	169 (2)
O1—H1E···O2^iv^	0.82 (3)	1.88 (3)	2.6937 (17)	176 (3)
C1—H1B···O4^iii^	0.97	2.60	3.479 (2)	151

Symmetry codes: (ii) *x*, −*y*, *z*−1/2; (iii) *x*, *y*, *z*−1; (iv) *x*, −*y*+1, *z*−1/2.
